# Metformin increases chemo-sensitivity via gene downregulation encoding DNA replication proteins in 5-Fu resistant colorectal cancer cells

**DOI:** 10.18632/oncotarget.17798

**Published:** 2017-05-11

**Authors:** Sung-Hee Kim, Soon-Chan Kim, Ja-Lok Ku

**Affiliations:** ^1^ Laboratory of Cell Biology, Cancer Research Institute, Seoul National University College of Medicine, Seoul 03080, Republic of Korea; ^2^ Department of Biomedical Sciences, Seoul National University College of Medicine, Seoul 03080, Republic of Korea

**Keywords:** colorectal cancer, 5-Fu resistant-cell line, metformin, cancer stem cell (CSC), DNA replication

## Abstract

Metformin is most widely prescribed for type 2 diabetes. Recently, evidences have shown that metformin has anticancer effects on pancreatic-, colorectal-, ovarian-, and other cancers. Because metformin has less adverse effects and is inexpensive, it could be a useful chemo-therapeutic agent with anticancer effects. In this study, we demonstrated metformin inhibited by cell proliferation, cell migration ability, clonogenic ability, and cancer stem cell population. Metformin also induced cell cycle arrest in parental-(SNU-C5), and 5-Fu resistant-colorectal cancer cell line (SNU-C5_5FuR). Moreover, a treatment that combines 5-Fu and metformin was found to have a synergistic effect on the cell proliferation rate, especially in SNU-C5_5FuR, which was mediated by the activation of AMPK pathway and NF-ƙB pathway, well-known metformin mechanisms. In this study, we suggested novel anticancer mechanism of metformin that inhibited DNA replication machinery, such as the MCM family in SNU-C5_5FuR. In conclusion, we provided that how metformin acts as not only a chemo-sensitizer, but also as a synergistic effector of 5-Fu in the 5-Fu resistant-cell line. We speculate that metformin used for adjuvant therapy is effective on 5-Fu resistant cancer cells.

## INTRODUCTION

Colorectal cancer is the third most diagnosed cancer with an annual estimated death of 60,000 [1]. 5-Fluorouracil (5-Fu) is the standard chemotherapeutic agent in colorectal cancer that acts as an antimetabolite drug through thymidylate synthase (TS) inhibition and incorporated into nucleic acid, DNA, and RNA. Despite the usage frequency, 5-Fu has low effectiveness in colorectal cancer at about 10 to 15% [2]. Recently, combination therapies with other drugs like irinotecan and oxaliplatin are suggested to improve 5-Fu effectiveness using the independent activity pathway. Nevertheless, 5-Fu still has low effectiveness due to drug resistance. Furthermore, there are diverse mechanisms related to 5-Fu resistance like changes in drug uptake and/or catalytic enzyme activities [3, 4].

Metformin (N’, N’-dimethylbiguanide) was developed as a type 2 diabetic therapeutic agent [5]. Unlike the other biguanides, buformin and phenformin, metformin is the most frequently prescribed drug due to minimized toxicity and side effects [5, 6]. Interestingly, there are some studies that state metformin is related to anticancer effects: metformin significantly decreased the incidence risk of pancreatic-, colorectal-, and ovarian cancers [7–10], as well as various cancer cell lines [11–15]. Furthermore, metformin influences apoptosis and cell cycle arrest, which reduces cancer cell populations [16, 17]. The mechanism of metformin action is well-studied by working through pivotal AMPK/mTOR pathways [18]. Metformin activates AMPK after LKB1 and sequentially inactivates mTOR. Along this pathway, p53 is activated according to autophagy and decreased protein synthesis. The cell cycle is arrested as a result, which means that metformin mechanism is related to the chemotherapeutic effect. Moreover, in SW620 colon cancer cell line, metformin affects cell proliferation, apoptosis, and cell cycle via selectively targeted CD133+ cancer stem cell populations [19].

In this paper, we showed metformin selectively affecting cell proliferation and metastatic behavior on 5-Fu resistant-colorectal cancer cell lines caused by the inhibition of DNA replication machinery.

## RESULTS

### Metformin reduced cell proliferation and increased G_1_ arrest in colon cancer cell lines

**Figure 1:**
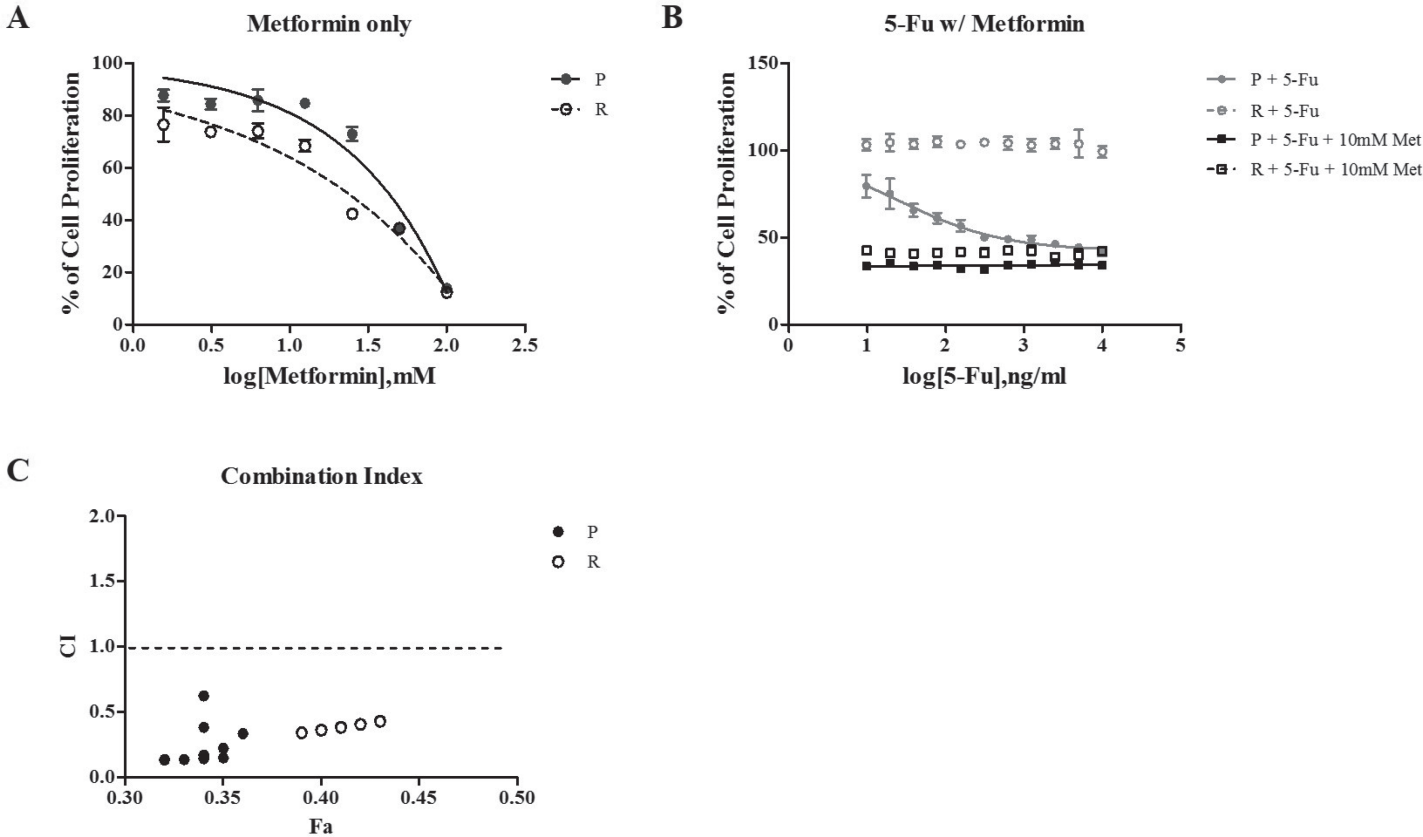
The relative cell proliferation rate as treated with 5-Fu, metformin, and combination 5-Fu with metformin Metformin only treated with serial dilution from 100 mM (**A**) 5-Fu only or both 5-Fu and 10 mM Metformin (**B**). 5-Fu was treated with serial dilution from 10 μg/mL. The cell proliferation rate was confirmed after a 72 h treatment of drugs using Ez-Cytox, which assessed NADH-dehydrogenase in live cells. (**C**) represents the combination index (CI) that is calculated using Compusyn (http://www.combosyn.com/). CI < 1 means the synergistic effect of combination 5-Fu and metformin treatment, CI = 1 is an additive effect, and CI > 1 is the antagonistic effect. The assay was performed three times. P is the parental cell line, SNU-C5 and R are the resistant cell lines, SNU-C5_5FuR and 5-Fu are the serial dilution of 5-Fu, and Met is the metformin.

Recently, there have been some reports that metformin inhibits cell proliferation and induces cell cycle arrest [16]. Based on these studies, we investigated if metformin affects cell proliferation and the cell cycle in parental-(SNU-C5) or 5-Fu resistant colorectal cancer cell lines (SNU-C5_5FuR). To confirm the metformin effects on cell proliferation, we tested 3 different conditions: 5-Fu serial dilution treatment from 10 μg/mL, metformin serial dilution treatment from 100 mM, and combination treatment of 5-Fu serial dilution with 10 mM metformin. As shown in Figure 1, SNU-C5 was sensitive to 5-Fu since it was treated with a 5-Fu serial dilution. However, SNU-C5_5FuR was merely changed to 5-Fu, which indicated that the drugs react to parental and 5-Fu resistant cell lines. Both cell lines were affected by metformin, especially the 5-Fu resistant cell line. We confirmed the synergistic effect of combination treatment that leads to the serial dilution of 5-Fu and 10mM metformin. The proliferation rate of SNU-C5 and SNU-C5_5FuR cell lines was effectively reduced by the combination treatment of 5-Fu and metformin. Consequently, all combination doses had the synergistic effect (Figure 1C, combination index (CI) < 1). At the lowest 5-Fu dose and 10mM metformin combination, the proliferation rate decreased 57.5% in SNU-C5 and 60.2% in SNU-C5_5FuR, compared to the 5-Fu only treatment. At the highest 5-Fu dose and 10mM metformin combination, the proliferation rate decreased 17% and 57% in SNU-C5 and SNU-C5_5FuR, respectively. In this study, we suggested that metformin reduced cell proliferation and increased sensitivity to 5-Fu in SNU-C5_5FuR.

**Figure 2:**
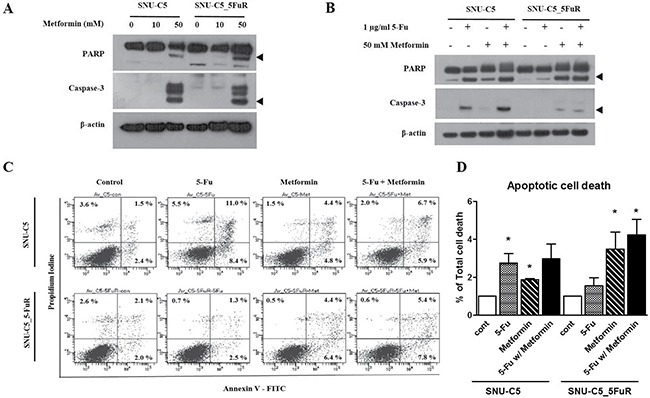
The expression levels of apoptotic pathway proteins as treated with various doses of metformin (**A**) and combination 1 μg/mL of 5-Fu and 50 mM of metformin treatment (**B**) by western blotting. The arrow head indicates the active form of PARP or caspase-3. Apoptotic cell death detected by Annexin V/PE staining as treated with 0.5 μg/mL of 5-Fu and 10 mM of metformin (**C**). (**D)** represents the percentage of Annexin V-positive population (*p < 0.05).

**Figure 3:**
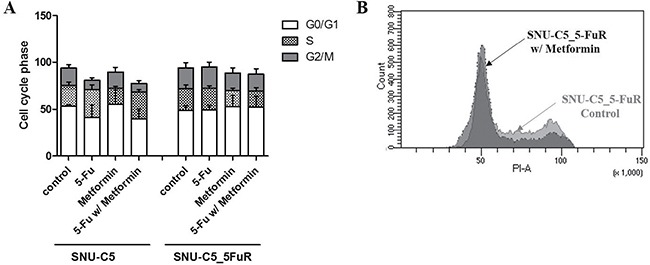
Cell cycle analysis of SNU-C5 and SNU-C5_5FuR when treated with 1 μg/mL of 5-Fu and 50 mM of metformin as well as combination 5-Fu and metformin treatment The bar graphs indicate the changes in the cell cycle progression (**A**) and raw data of cell cycle distribution in SNU-C5_5FuR cell lines (**B**). The assay was performed three times.

Next, we confirmed apoptotic proteins whether metformin leads to cell death or cell proliferation. By treating 50 mM of metformin, the cleaved caspase-3 and PARP were increased in a similar manner in both cell lines, SNU-C5 and SNU-C5_5FuR, when compared to lower dose (10mM) of metformin (Figure 2A). Therefore, we speculated that metformin induced cell death. In SNU-C5, the 5-Fu treatment and combination of 5-Fu and metformin treatment significantly increased the expression level of cleaved caspase-3 and PARP. In SNU-C5_5FuR, however, the apoptotic proteins were induced by metformin or 5-Fu and metformin combination treatment (Figure 2B). We also substantiated these results by analyzing Annexin V positive cells (Figure 2C, 2D). Thus, metformin decreased cell proliferation and increased cell death with induced apoptotic proteins. It also has higher synergistic effects with 5-Fu in SNU-C5_5FuR than SNU-C5. To confirm if metformin affects the cell cycle, we analyzed the 1 μg/mL of 5-Fu or 50mM of metformin treatment and 5-Fu and metformin combination treatment to SNU-C5 and SNU-C5_5FuR (Figure 3). The G_0_/G_1_ percentages in both SNU-C5 and SNU-C5_5FuR cell lines were increased to 21.25% and 30.07%, respectively, when treated with metformin. As the combination treatment to SNU-C5_5FuR, 27.23% of the cells were arrested at the G_0_/G_1_ phase, which means that metformin caused G_0_/G_1_ arrest. These results suggested that both 5-Fu and metformin influenced SNU-C5 in cell proliferation and death. In contrast, only metformin or combination 5-Fu and metformin treatment influenced SNU-C5_5FuR. Consequently, we speculated that metformin could have synergistic effects on 5-Fu.

### Metformin influenced cell migration, clonogenicity and angiogenesis

**Figure 4:**
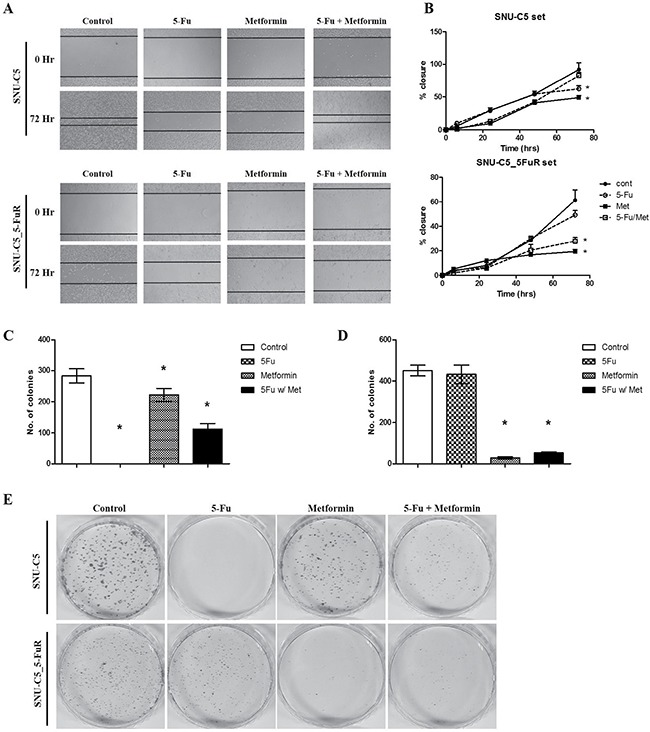
Metformin affected wound healing capacity and clonogenicity The wound healing assay and clonogenic assay were performed by 0.5 μg/mL of 5-Fu and 10 mM of metformin as well as combination 5-Fu and metformin treatment. For the migration assay, 5000 cells/well were seeded, wounded, and then treated with PBS (as control), 5-Fu, and metformin. The wound was observed at 0, 6, 24, 48, and 72 h. (**A)** represents the taken phase-contrast picture images at 0 and 48 h. (**B)** shows the calculated cell migration where the black closed circle is control, open circle is 5-Fu treatment, closed square is metformin, and open square is combination treatment. For clonogenic assay, 0.5 × 10^3^ cells are pre-treated by 5-Fu w/ or w/o metformin and seeded in a 60 mm dish. After 14 days, the colonies are counted by staining with crystal violet. The experiments are performed three times (*p < 0.05). (**C** and **D)** represent the number of SNU-C5 and SNU-C5_5FuR coloines, respectively (*p < 0.05). (**E)** shows the picture images of those colonies. The assay was performed three times.

To investigate the metformin effects on cell migration and clonogenic ability, we performed wound healing and clonogenic assays. 0.5 μg/mL of 5-Fu and 10 mM of metformin, and the combination treatment of 5-Fu and metformin were treated to SNU-C5 and SNU-C5-5FuR cell lines, respectively. After 0, 6, 24, 48, and 72 h, we confirmed the relative cell migration rate. As shown in Figure 4A and 4B, both 5-Fu and metformin influenced the cell migration rate. Compare to SNU-C5 control, the migration rate decreased at 38.78% and 51.65% when treated with 5-Fu and metformin, respectively. It was also decreased 19.51% due to the combination treatment of 5-Fu and metformin in SNU-C5 parental cell line. For SNU-C5_5FuR, the migration rate decreased 27.78%, 72.95%, and 61.04% when treated with 5-Fu, metformin, and combination, respectively. SNU-C5_5FuR cell line tended to delayed migration when compared with SNU-C5. The two cell lines had different cell migration rates when treated with drugs. SNU-C5 was more influenced by 5-Fu than metformin, while SNU-C5_5FuR was more sensitive to metformin. The cell migration capacity has influenced metformin more than 5-Fu in this cell line. The data showed that metformin might influence cell migration and that was effective in targeting 5-Fu resistant cancer cell line. Metformin also inhibits metastatic behavior like angiogenesis in many cancers [20, 21].

The clonogenic ability was comparable with cell migration patterns when treated with drugs: SNU-C5 was more affected by 5-Fu than metformin. Metformin treatment and combination of 5-Fu and metformin effectively reduced clonogenic ability in SNU-C5_5FuR cell lines. (Figure 4C, 4D).

To investigate metformin on angiogenesis, we also confirmed HIF-1α and VEGF. We found that HIF-1α expression was decreased when treated with 5-Fu in SNU-C5 and with metformin in SNU-C5_5FuR. As a result, we suggested SNU-C5_5FuR is more sensitive to metformin than SNU-C5. Additionally, metformin affected cell migration ability and expression of angiogenesis related proteins.

### Metformin's effect on AMPK/mTOR axis and NF-ƙB pathway

**Figure 5:**
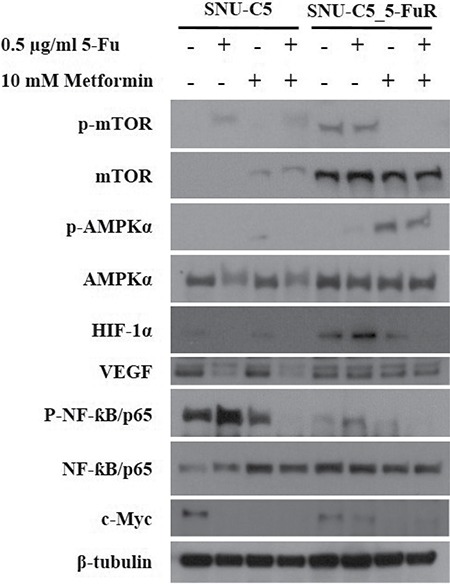
Metformin on AMPKα/mTOR axis pathway and NF-ƙB pathway effector proteins AMPK/mTOR signaling pathway is well-known mechanism of metformin. Total AMPK and mTOR, and their phosphorylation form were determined by western blot analysis when treated with 5-Fu and/or metformin. Also, NF-ƙB and angiogenesis related related proteins, HIF-1α, VEGF were confirmed. β-tubulin was used an a internal control.

The well-known metformin mechanism was via the AMPK/mTOR axis that inhibits cellular metabolism and protein synthesis by metformin [18]. Metformin activates the AMPK pathway, which inhibits mTOR. In addition, the NF-ƙB pathway is known to affect metformin [22]. To confirm the metformin action pathway, we verified protein levels by western blot analysis. As shown in Figure 5, phospho-AMPKα increased and phospho-mTOR decreased when treated with metformin, especially in SNU-C5_5FuR cell line. In contrast, no phospho-AMPKα augmentation was detected in SNU-C5 cell line. The NF-ƙB pathway decreased when treated with a combination of the 5-Fu and metformin in both cell lines as opposed to a single treatment of 5-Fu. In this study, we confirm that metformin inhibits cell proliferation and migration via the AMPK/mTOR axis and NF-ƙB pathway. In addition, the SNU-C5_5FuR cell line is more sensitive to metformin than SNU-C5.

### Metformin influenced cancer stem cell population and tumor sphere formation

**Figure 6:**
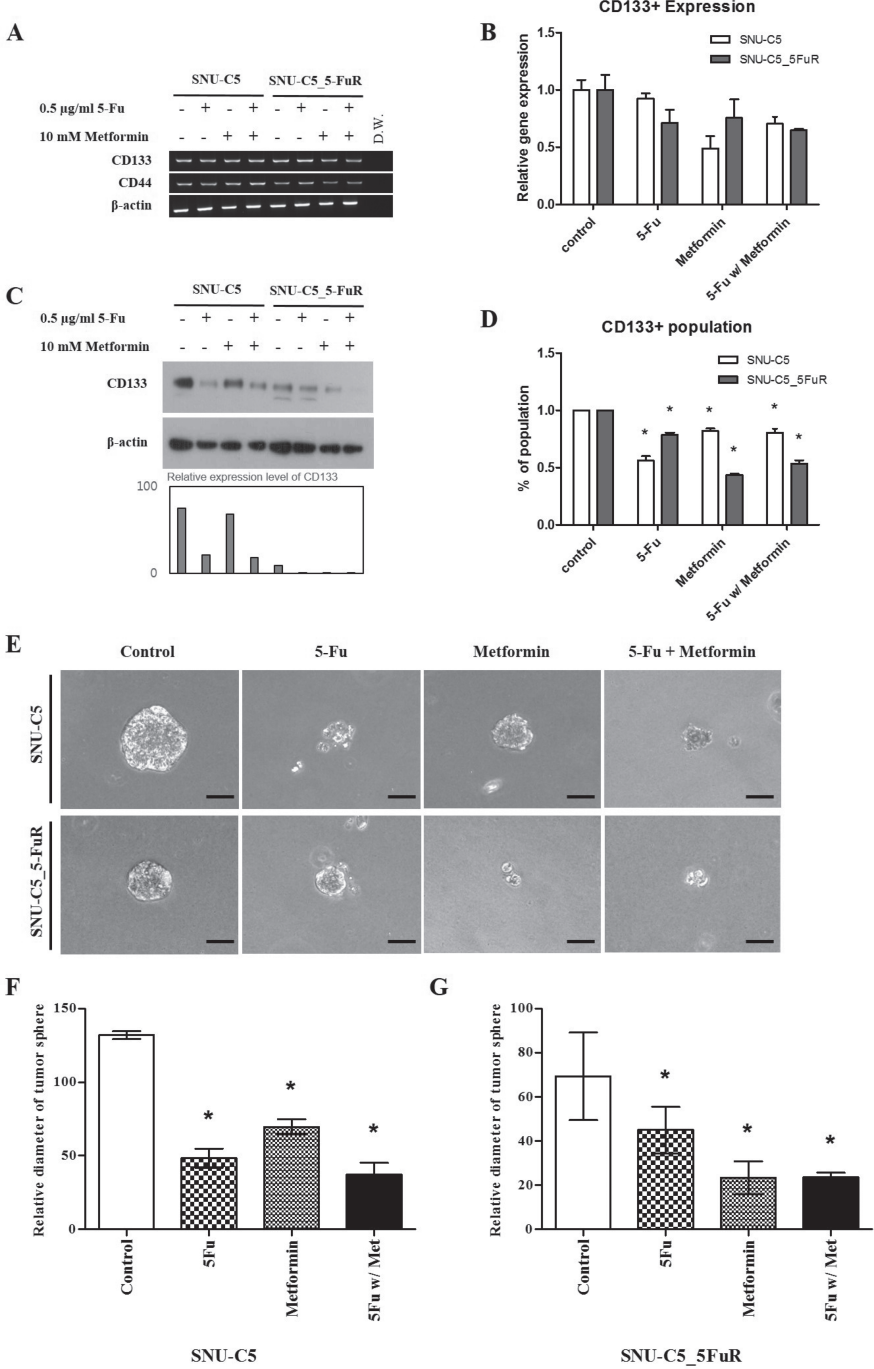
Cancer stem cell marker (CD133) expression levels treated with 0.5 μg/mL of 5-Fu and 10 mM of metformin by RT-PCR (**A**) quantitative-RT-PCR (**B**) western blot analysis (**C**) and FACs analysis (**D**) (*p < 0.05). Quantitative RT-PCR data was normalized by β-actin expression level and then calculated ddCt value using 7300 System SDS v1.4. Software. Western blot band intensities were calculated by ImageJ (the graph) in the lower bar graph. After 3D culture for tumor sphere formation with RGF-BME, microscopical analysis (magnification × 250, Scale Bar = 50 μm) (**E**). (**F** and **G)** represent relative diameter of tumor sphere measured using Image J (*p < 0.05). All of the experiments were performed three times.

Recently, rising evidences suggest that cancer stem cells (CSCs) are related to drug resistance in cancers [23, 24]. Based on these theories, we investigated the changes in cancer stem cell population expressed CD133 and CD44, which are well known colorectal cancer stem cell surface markers caused by 5-Fu, metformin, and combination treatments. In SNU-C5 and SNU-C5_5FuR, 5-Fu and metformin reduced the transcriptional and translational expression levels of CD133 (Figure 6A–6C). In SNU-C5, 71.8%, 15.0%, and 75.3% of the CD133 protein expression decreased when treated with 5-Fu, metformin, and combination, respectively. In SNU-C5_5FuR, 87.9%, 90.1%, and 93.4% decreased when treated with 5-Fu, metformin, and combination, respectively. We performed fluorescence-activated cell sorter (FACS) analysis (Figure 6D) and confirmed tumor sphere formation (Figure 6E–6G) to verify the above data. As shown in Figure 6, SNU-C5 was more influenced by 5-Fu and SNU-C5-5FuR influenced by metformin. We suggest that metformin influences cancer stem cell populations according to these results.

### Metformin reduced DNA replication machinery genes in 5-Fu resistant cancer cell line

**Figure 7:**
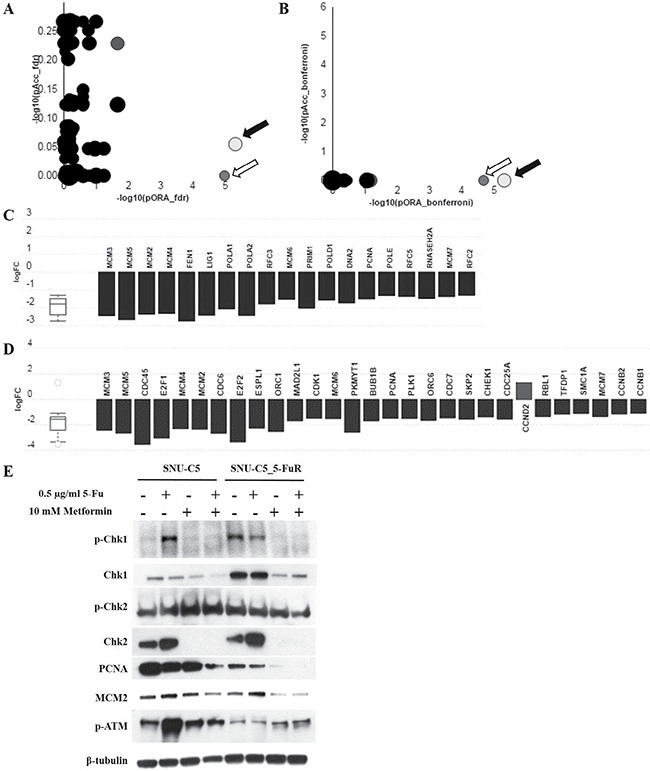
Metformin reduced the expression level of DNA replication machinery and mitotic cell cycle genes As a result of RNA seq., DNA replication (KEGG: 03030) (black arrows in **A** and **B**), and mitotic cell cycle (KEGG: 04110) (white arrows in A and B) genes were downregulated when treated with metformin. A is the gene correction by False Discovery Rate (FDR) and B is by Bonferroni correction. All the genes from DNA replication pathway (**C**) and mitotic cell cycle pathway (**D**) were represented in terms of their measured fold change (y-axis) and accumulation (x-axis). The related protein level was detected by western blot analysis (**E**).

To investigate the other mechanisms of metformin action on 5-Fu resistant cancer cell line, we performed RNA sequencing Figure 7A, 7B. After conducting RNA-seq, we sorted genes that have more than two fold changes and have *p*-values less than 0.05, as determined in accordance with the absolute value of RPKM and reads quality. A total of 658 genes from 25,269 genes were sorted. By using iVariantGuide software, pathways affected by metformin treatment in RNA levels were detected. As shown in Figure 7C and 7D, DNA helicase activity was mostly affected while DNA replication machinery and cell cycle regulation genes were significantly reduced. We also verified protein expression levels related to DNA replication machinery: MCM2 and PCNA (Figure 7E). Interestingly, MCM2 and PCNA were reduced more when SNU-C5_5FuR was treated with metformin rather than SNU-C5. Chk1 and Chk2 are essential proteins for DNA damage response and cell cycle check point, which were reduced in metformin treated parental-, and resistant cancer cell lines. Phospho-Chk1 was reduced when treated with metformin, however, phospho-Chk2 merely changed in both cell lines. In summary, metformin selectively affected the DNA damage response and DNA replication, especially in 5-Fu resistant colon cancer cell line.

## DISCUSSION

The key research question of this study was to determine if metformin has anticancer effects and if it acts as a chemosensitizer for recovering chemo-resistance in colorectal cancer line, SNU-C5, and 5-Fu resistant cell line.

We first performed cell proliferation assays and cell cycle analysis after treating 5-Fu or metformin and combination 5-Fu and metformin treatment to SNU-C5 and SNU-C5_5FuR. As shown in Figure 1, the SNU-C5 cell line was more sensitive than SNU-C5_5FuR at different 5-Fu concentrations. When treated with different 5-Fu doses, the proliferation rate of the resistant cell line did not significantly change. On the contrary, the parental cell line decreased. At 10 μg/mL of 5-Fu, the highest dose, cell proliferation decreased about 39% in SNU-C5. Both cell lines, SNU-C5, and SNU-C5_5FuR, decreased cell proliferation affecting metformin in treatment (Figure 1A). Thus, we wonder if metformin and 5-Fu has synergistic effects. For the combination treatment, serial dilution from 10 μg/mL of 5-Fu and 100 mM metformin were treated to SNU-C5, and SNU-C5_5FuR. Combination treatment has synergistic effects on reducing cell proliferation at every concentration of 5-Fu for both SNU-C5 and SNU-C5_5FuR, as confirmed by CI value calculation (< 1) (Figure 1C). According to Chen Qu. *et al*, metformin has reversal effects on chemo-resistance in breast cancer cells [25]. Corresponds to these result, our data suggested that the 5-Fu resistant-cell line, SNU-C5_5FuR, was more sensitive with metformin than the parental cell line. The increased expression level of apoptotic proteins was validated these data. As shown in Figure 2B, the active form of PARP and caspase-3 expressed with cell death were mainly induced by 5-Fu in SNU-C5 and metformin in SNU-C5-5FuR.

Our data showed that metformin reduces cell proliferation via increased sensitivity to 5-Fu and apoptotic protein expression, especially in 5-Fu resistant-colorectal cancer cell lines. We observed metformin affecting the cell cycle, migration rate, and clonogenicity as in the preceding data. In SNU-C5_5FuR cells, treatment with 5-Fu alone had less impact on cellular behavior like the cell cycle. In contrast, treatment with metformin and combination increased cell death, cell cycle arrest at G_0_/G_1_ phase, and decreased rate of migration and clonogenicity. The metformin effects had a discrepancy between parental cell line and 5-Fu resistant cell line. SNU-C5 was less sensitive to metformin than 5-Fu. With these results, we suggested the selective response of metformin to 5-Fu resistant cell lines. In addition, metformin has synergistic effects with 5-Fu (Figure 4). The VEGF expression level was enhanced by of the cooperation between c-Myc and HIF-1α [26], while the expression of c-Myc was regulated by microRNA-33a from metformin treatment [27]. HIF-1α is a key regulator of cancer metabolism particularly in anaerobic condition. In addition, both of HIF-1α and VEGF are involved in angiogenesis, which is an important feature of cancer metastasis for cellular behavior. However, expression of HIF-1α, and VEGF is merely regulated by metformin therefore metformin is less influence to cancer metastatic proteins. To date, there has been clinical trials of metformin for chemoprevention with angiogenic effect in Barretts's metaplasia, colorectal adenoma, and prostate cancer [20]. In addition to our data, metformin has been suggested to have anti-angiogenic effects. Furthermore, there is evidences that metformin regulates the cell cycle by G_0_/G_1_ arrest and cell death via AMPK pathway. When treated with metformin, phospho-AMPK increased in SNU-C5_5FuR cell while phospho-mTOR was decreased (Figure 5). This confirmed that the activated AMPK signaling pathway might play a key role in the 5-Fu resistant cell line. Metformin suppresses MDR1 expression in the concomitant inhibiting nuclear factor ƙ-light-chain-enhancer of activated B cells (NF-ƙB) [22, 28, 29]. Our data shows that metformin inhibited NF-ƙB pathway and the combination treatment of 5-Fu and metformin has significantly affected the activation. However, used cell lines SNU-C5 and SNU-C5_5FuR did not expressed MDR1 (data are not shown).

Besides the AMPK-mTOR axis and NF-ƙB pathway, we wondered if the other metformin pathways acted on 5-Fu resistant cancer cells. Therefore, we confirmed the cancer stem cell population from the evidences that metformin inhibits cancer cell proliferation via targeting cancer stem cell population [19, 30, 31]. CD133, also known as prominin-1 or AC133, is a 7-transmembrane surface maker of cancer stem cells, especially in colon cancer [32]. We observed that metformin affects the clonogenicity capacity, as shown in Figure 4E. This may demonstrated that metformin is related to the self-renewal ability with the stemness characteristic The CD133 positive cell population and expression level of CD133 was reduced in SNU-C5 and SNU-C5_5FuR, and these were related with tumor sphere formation (Figure 6). Notably, CD133 expression and ability of tumor sphere formation in SNU-C5_5FuR was significantly reduced by metformin and combination treatment of 5-Fu and metformin.

Additionally, we performed RNA sequencing using SNU-C5_5FuR control and metformin treated samples to confirm the metformin regulated pathway in the 5-Fu resistant cell line. As a result, we confirmed DNA polymerization complex genes, such as MCM2, PCNA, and cell cycle related genes, that were depleted by metformin treatment in RNA and protein level in 5-Fu resistant cancer cell line. The MCM complex is a major component in eukaryotic DNA replication machinery, which interacts with Chk1 as DNA becomes damaged [33]. Consequently, metformin was reported to damage DNA, activated the intracellular ATM/Chk2 checkpoint, and regulation the cell cycle [34]. MCM2, PCNA, and Chk1 were down regulated and ATM was activated by metformin in the SNU-C5_5FuR cell line. Therefore, the previous cell cycle arrest data increased 5-Fu sensitivity and induced metformin in 5-Fu resistant cell line due to DNA damage. Interestingly, there was less influence on the MCM2 and PCNA expression level due to metformin treatment on the SNU-C5 parental cell line, which means that metformin selectively affects DNA replication. This is particularly present in the SNU-C5_5FuR cell line.

In conclusion, metformin has shown synergistic effects in combination with the conventional chemotherapeutic agent, 5-Fu through DNA damage and inhibition of DNA replication machinery, particularly in 5-Fu resistant cancer cell line. Therefore, we speculate that metformin could be used in adjuvant chemotherapeutics without severe side effects.

## MATERIALS AND METHODS

### Cell culture and chemicals

SNU-C5, a human colorectal cancer cell line, was obtained from the Korean Cell Line Bank (KCLB, Seoul, Korea). SNU-C5_5FuR, a 5-fluorouracil-resistant population of SNU-C5, was established as previously described [35, 36] by treatment with 4000 ng/mL of 5-Fu (Sigma- Aldrich, MO, USA). Cell lines were cultured in RPMI1640 (Thermo-Fisher scientific, MA, USA) medium supplemented with 10% fetal bovine serum and 1.1% penicillin/streptomycin.

Metformin and 5-Fu were purchased from Sigma-Aldrich and were dissolved in deionized water and DMSO, respectively.

### Cell proliferation, migration, clonogenic assay, and tumor sphere formation

50,000 cells/well were seeded in a 96-well plate and incubated for 16 h before being treated 5-Fu and/or metformin for a proper amount of time. The cell proliferation rate was assessed by NADH-dehydrogenase in the live cell using Ez-Cytox (Daeillab service, Seoul, Korea). The absorbance at 450 nm was measured by a MULTISKAN FC Microplate Photometer (Thermo-Fisher Scientific). The assay was performed three times while.

50,000 cells/well were seeded on a 24-well plate with wound-healing insert (Cell biolabs, CA, USA) and incubate for overnight for a cell migration assay. The insert was carefully removed and incubated with cell culture medium containing 5-Fu and/or metformin. 400 × magnification images were taken using a CCD camera (Olympus, Tokyo, Japan).

For the clonogenic assay, 50,000 cells/well were seeded on a 6-well plate. After incubation overnight, cells were exposed to 5-Fu and/or metformin for 72 h, then trypsinized cells were re-seeded on a 60-mm culture dish with 5 × 10^3^ cells. The cells were then incubated for 14 days in humidified incubators. Colonies were fixated with methanol and acetic acid in a 1:7 solution for 10 min at RT and staining with 0.5% crystal violet for 1 h at RT. The colonies were counted after a water wash and air drying.

Tumor sphere formation was performed using reduced growth factor (RGF)-basement membrane extract (BME) (Thermo-Fisher scientific) with 50,000 cells/well of cells on a 24-well plate, then treated with 5-Fu and/or metformin.

### Apoptotic analysis

To detect apoptotic cell death, we used an Annexin V-FITC/PE staining kit (Invitrogen, CA, USA) after 5-Fu and metformin treatment. Cells were resuspended to 100 μl per sample of 1X Annexin-binding buffer with 5 μl of Annexin V-FITC, and 1 μl of 100 μg/mL propidium iodide (PI) (Sigma-Aldrich) solution. After incubation at RT for 15 min, the cells were analyzed by flow cytometry (BD, NJ, USA).

### Cell cycle analysis

To analyze the cell cycle distribution, cells were fixed overnight in 70% ethanol at −20°C. The cellular DNA was stained with 100 μg/mL of PI for 30 min on ice and analyzed using a flow cytometry based on DNA content.

### Reverse transcriptase (RT) - PCR and real-time quantitative (q) RT-PCR

cDNA was synthesized using easy-BLUE^TM^ kits (Intron biotechnology, Gyeonggi, Korea) and a Quantitect Reverse transcription kit (Qiagen, Hilden, Germany) according to manufacturer's instructions. The PCR mixture contained 1 μl of 100 ng/μl cDNA, 10 × buffer, 2.5 mM of dNTP, 0.1pM of primers and 1 unit of Taq DNA polymerase (Intron biotechnology). PCR was performed on a thermal cycler (PCR System 9700, Applied Biosystems; CA, USA).

The qRT-PCR was performed using 2X SYBR on a 7300 instrument (Applied Biosystems). Each reaction was performed three times on a 96-well plate. Raw Ct was calculated with 7300 system version 1.4.0. The used oligonucleotide primer sequences are listed in Supplementary Table 1.

### Western blot analysis

Whole cell lysates were prepared by RIPA kits (Atto, Tokyo, Japan) while the protein concentration was accomplished by a SMART^TM^ micro BCA kit (Intron biotechnology). Proteins were separated on Bis-Tris pre-cast gels and transferred to PVDF membrane (Bio Rad, CA, USA). Primary antibodies against PARP (BD), caspase-3, CD133, VEGF, Chk1, p-Chk1, Chk2, p-Chk2, PCNA, MCM2, p-ATM (Abcam, Cambridge, UK), mTOR, p-mTOR, AMPKα, p-AMPKα (Cell signaling, MA, USA), HIF-1α (Sigma-Aldrich), β-actin, and β-tubulin (Santa Cruz, CA, USA) were used at 1:1000. Peroxidase conjugated secondary antibody were against rabbit or mouse (Jackson Immunoreasearch, MD, USA) diluted 1:5000. Chemiluminescent solution was obtained from Intron biotechnology and detected using Fuji RX film.

### Immunophenotyping

FcR blocking reagents and CD133-PE antibodies (Miltenyi Biotec, Bergisch Gladbach, Germany) were added to cells and incubated for 10 min at 4°C. The CD133 expression was detected by flow cytometry.

### RNA sequencing

The total RNA was isolated from cell lysate using Trizol and RNeasy Kit (Qiagen) according to manufacturer's protocol. The sequencing libraries were prepared using the Illumina TruSeq Stranded Total RNA Library Prep Kit (Illumina, San Diego, USA). Following base-calling and alignment with the Tuxedo Suite, rejected reads were analyzed with default parameters for RNA and aligned to the reference genome (human hg19) by Tophat (v2.0.13). The output was filtered to include at least one rescued read and two unique seed reads as well as exclude known, recurrent artifacts. The aligned results were added to Cuffdiff (v2.2.0) to report differently expressed genes. Geometric and blind methods were applied to normalize the library and estimate the dispersion. The exon RPKM of metformin treated SNU-C5_5FuR sample was compared to the exon RPKM of SNU-C5_5FuR control sample. The detailed methods are described in Supplementary Methods.

## SUPPLEMENTARY MATERIALS TABLES


