# Supplementary material for: Metformin increases chemo-sensitivity via gene downregulation encoding DNA replication proteins in 5-Fu resistant colorectal cancer cells
Source: Oncotarget. 2017 May 11;8(34):56546–57. doi: 10.18632/oncotarget.17798 (PMC5593582; doi:10.18632/oncotarget.17798)
Supplement: Supplementary file 1 [file oncotarget-08-56546-s001.pdf]

Metformin increases chemo-sensitivity via gene downregulation encoding DNA replication proteins in 5-Fu resistant colorectal cancer cells

Supplementary Materials

MATERIALS AND METHODS

mRNA sequencing

RNA purity was determined by assaying 1 µl of total RNA extract on a NanoDrop8000 spectrophotometer. Total RNA integrity was checked using an Agilent Technologies 2100 Bioanalyzer with an RNA Integrity Number (RIN) value.

mRNA sequencing libraries were prepared according to the manufacturer's instructions (Illumina Truseq stranded mRNA library prep kit). mRNA was purified and fragmented from total RNA (0.8ug) using poly-T oligo-attached magnetic beads using two rounds of purification. Cleaved RNA Fragments primed with random hexamers were reverse transcribed into first strand cDNA using reverse transcriptase, random primers, dUTP in place of dTTP. (The incorporation of dUTP quenches the second strand during amplification, because the polymerase does not incorporate past this nucleotide.) These cDNA fragments then had the addition of a single 'A' base and subsequent ligation of the adapter. The products were purified and enriched with PCR to create the final strand specific cDNA library. The quality of the amplified libraries was verified by capillary electrophoresis (Bioanalyzer, Agilent).

After qRT-PCR using SYBR Green PCR Master Mix (Applied Biosystems), we combined libraries that index tagged in equimolar amounts in the pool. Cluster generation occurred in the flow cell on the cBot automated cluster generation system (illumina). And then the flow cell loaded on HISEQ 2500 sequencing system (Illumina), performed sequencing with 2 × 100 bp read length.

RNASeq analysis method

Mapping reads on a reference genome and calculating expression between samples

At first, reads for each sample were mapped to the reference genome (human hg19) [1] by Tophat (v2.0.13) [2]. The aligned results were added to Cuffdiff (v2.2.0) [3] to report differentially expressed genes. For library normalization and dispersion estimation, geometric and blind ("blind" when each condition has single replicates, or "pooled" when multiple replicates (more than one sample) are available)methods [3] were applied.

Identification of DEGs and functional enrichment analysis

Cuffdiff provides various output files, and using one of its outputs, "gene_exp.diff", DEGs (Differentially Expressed Genes) were identified. To detect DEGs between sample1 as control and sample2 as case, two filtering processes were applied. First, using Cuffdiff status code, genes that only have "OK" status were extracted. Status code indicates whether each condition contains enough reads in a locus for a reliable calculation of expression level, and "OK" status means the test is successful to calculate gene expression level. For the second filtering, 2 fold change was calculated and only genes belonging to the following range were selected.

Up-regulated: log2[case]-log2[control] > log2(2) = 1

Down-regulated: log2[case]-log2[control] < log2 (1/2) =–1

For ontology analysis, genes after 2 fold change were picked and applied to DAVID [4] as an input to get a comprehensive set of functional annotation. Disease, Gene ontology, pathway categories were selected, and Ease score was changed from 0.1 to 1 to include more output. Ease score is a conservative adjustment to the Fisher exact probability. It weights significance in favor of the association supported by more genes. DAVID then generates functional annotation chart which lists annotation terms and their associated genes under study.

REFERENCES

1. http://support.illumina.com/sequencing/sequencing_software/igenome.html.
2. http://ccb.jhu.edu/software/tophat/.
3. http://cole-trapnell-lab.github.io/cufflinks/cuffdiff/
4. http://david.abcc.ncifcrf.gov/.

Supplementary Table 1: List of primers [1, 2]
F: forward primer; R: reverse primer

Gene	Primer	Sequence
**CD133**	F	5′-CTGGGGCTGCTGTTTATTATTCTG-3′
	R	5′-ACGCCTTGTCCTTGGTAGTGTTG-3′
**CD44**	F	5′-GTGATCAACAGTGGCAATGG-3′
	R	5′- GGGCCCTAATTTCAGAAAGC-3′
**β-actin**	F	5′-GAGACCTTCAACACCCCAGC-3′
	R	5′-GCTCATTGCCAATGGTGATG-3′

REFERENCES

1. Kim SH, Kim KH, Yoo BC, Ku JL. Induction of LGR5 by H2O2 treatment is associated with cell proliferation via the JNK signaling pathway in colon cancer cells. Int J Oncol. 2012; 41:1744–50. doi: 10.3892/ijo.2012.1596.
2. Jeon YK, Kim SH, Choi SH, Kim KH, Yoo BC, Ku JL, Park JG. Promoter hypermethylation and loss of CD133 gene expression in colorectal cancers. World J Gastroenterol. 2010; 16:3153–60. doi:
